# Who is at risk of endometrial cavity breach at laparoscopic myomectomy?

**Published:** 2020-01-24

**Authors:** K Rajah, M Dizdar, N Balachandren, K Kriedt, E Saridogan, D Mavrelos

**Affiliations:** Reproductive Medicine Unit, University College Hospital, London, UK.

**Keywords:** Fibroids, subfertility, laparoscopic myomectomy, ultrasound, cavity breach

## Abstract

**Background:**

Submucous and large intramural fibroids cause heavy menstrual bleeding and can negatively impact reproductive outcomes. Large submucous and non-cavity distorting fibroids need to be removed laparoscopically. One of the risks of a laparoscopic myomectomy is breaching the endometrial cavity and there have been suggestions that this increases the risk of intrauterine adhesions. The aim of this study was to examine the role of various demographic and pre-operative ultrasound variables at predicting the risk of endometrial cavity breach during laparoscopic myomectomy.

**Methods:**

This was a retrospective study of women who underwent a laparoscopic myomectomy. Women who had more than one fibroid removed and women who did not have pre-operative ultrasound images available were excluded. The size of the fibroid, minimum distance from the endometrial cavity, surface area, intra-cavity surface area, protrusion ratio and extra-cavity size as well as the women’s age, parity and pre-operative GnRH analogue use were recorded. The outcome measure was the breach of the endometrial cavity at myomectomy. Univariate analysis was performed to identify variables that are associated with a cavity breach. A logistic regression analysis was used to identify the most significant predictor of a breach.

**Results:**

A total of 66 women were included in the study. From these, 10 women sustained a cavity breach. All pre-operative ultrasound variables, i.e. minimum distance of the fibroid from the cavity (p=0.001), protrusion ratio (p=0.001), total surface area (p=0.020), intra-cavity surface area (p=0.001), size (p=0.030) and extra-cavity size (p=0.001) were statistically different between the group that had a cavity breach and the group that did not. In a logistic regression model, protrusion ratio was selected as the best predictor of a breach (OR 1.22; 95% CI 1.10 – 1.37). All breaches occurred in women who were not given GnRH analogue.

**Conclusion:**

Identifying patients at increased risk of a cavity breach facilitates better individualized pre-operative counselling regarding the risk of a breach and the possibility of intrauterine adhesions. It will also trigger more intra-operative vigilance to minimize the risk of breaching the cavity and, subsequently, the risk of intrauterine adhesions if a breach does occur.

## Introduction

Uterine fibroids are common in women of reproductive age (Walker and Stewart, 2005). Depending on their size and relation to the endometrial cavity, fibroids can contribute to symptoms such as menorrhagia, and difficulty with conception (Stewart and Nowak, 1996). Submucous fibroids tend to be particularly symptomatic, causing heavy and irregular bleeds. There is good evidence that submucous fibroids also make conception less likely (Pritts et al., 2009). Intramural fibroids can also contribute to menstrual problems, particularly when they reach a size over 50mm diameter (Wegienka et al., 2003). The impact of intramural fibroids on fertility remains a subject of controversy but recent reports suggest that intramural fibroids over 30mm in diameter are associated with a reduction in the chance of livebirth after embryo transfer (Christopoulos et al., 2017).

Symptomatic women with fibroids that are in or near the endometrial cavity are commonly offered myomectomy to alleviate their symptoms (Saridogan, 2016). A large proportion of these fibroids is treated hysteroscopically, as this is a less invasive procedure, with good results (Vercellini et al., 1999). However, with increasing fibroid size and decreasing protrusion into the cavity, the effectiveness of hysteroscopic resection decreases and thus a laparoscopic approach to myomectomy may be considered. One of the risks of laparoscopic myomectomy is breaching the endometrial cavity. In consequence, some suggest that such a breach increases the risk of intrauterine adhesions which in turn could impact future fertility.

Whilst preoperative ultrasonic assessment of fibroids is routine (Stewart, 2015), to this date, there are no published data on the preoperative ultrasonic characteristics of fibroids that would make an endometrial cavity more likely to be breached during the procedure. Such information would be useful for both, surgeons and patients, for counselling and informed consent. Thus, our aim is to examine preoperative ultrasonic characteristics of fibroids and their association with endometrial cavity breach during laparoscopic myomectomy.

## Materials and methods

### Ethical approval

This was a retrospective observational study conducted at the Reproductive Medicine Unit at University College Hospital, London. We sought advice from the Joint Research Office of University College London and University College London Hospital regarding ethical approval and were informed that formal ethics approval was not needed for this study under the condition that a patient’s identifiable data would not be seen by anyone outside the clinical care team.

### Patient inclusion

We searched our clinical database for all patients who underwent a laparoscopic myomectomy between December 2014 and October 2017. We excluded women who did not have a pre-operative transvaginal ultrasound. Also, women who had more than two fibroids removed were excluded as it would not have been possible to accurately attribute a cavity breach to one particular fibroid. We also excluded women if we were unable to obtain the pre-operative ultrasound images. As part of their initial investigation, women had an ultrasound scan performed by an experienced operator in a standardized manner by using a transvaginal ultrasound probe (Voluson E8, GE Medical Systems, Milwaukee, WI, USA). The technique for scanning in our unit has been previously described in Mavrelos et al.(2011). Briefly, the endometrium was identified, and all fibroids were measured in three orthogonal planes. The images were stored in a computerized database (PIA Fetal Database, version 5.6.16.917, Viewpoint Bildverarbeitung GmBH, Munich, Germany). All laparoscopic myomectomies were performed by four advanced laparoscopic surgeons using a standardized surgical technique previously described by Bean et al (2017).

**Figure 1:**
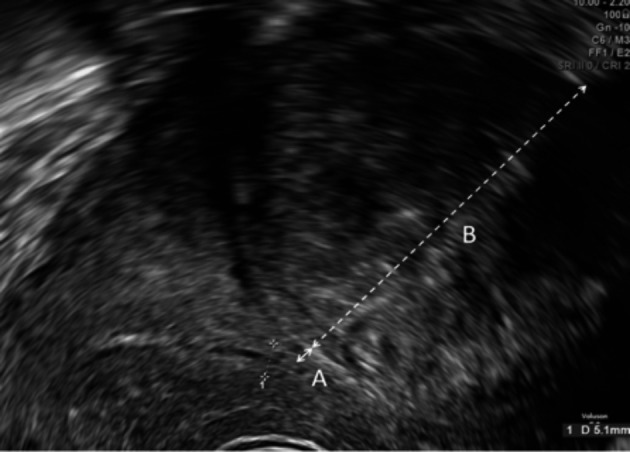
Ultrasound mage of an intramural/subserous fibroid showing the distance of the fibroid from the endometrial cavity (A) and the extra-cavity size (B).

**Figure 2:**
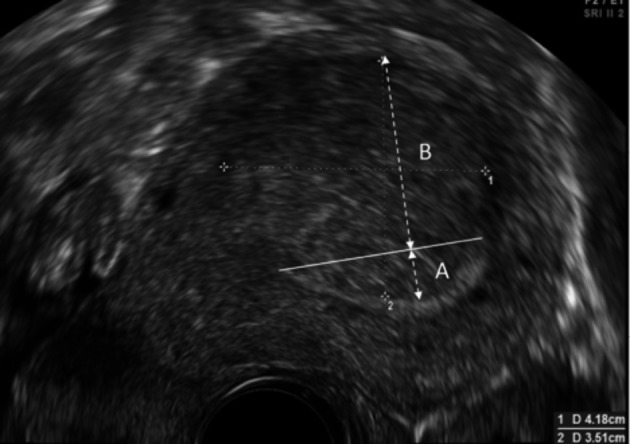
Ultrasound image of a submucous fibroid showing the subjective endometrial-myometrial junction (solid line), distance from the endometrial cavity (A) and extra-cavity size (B).

Two-dimensional ultrasound images of all women included in the study were reviewed by a single reviewer. The minimum distance of fibroids from the endometrial cavity was determined in the longitudinal view except for lateral fibroids when it was determined in the transverse view. For non-cavity distorting fibroids, we measured the distance from the point of the fibroid that was closest to the endometrial cavity in a perpendicular line (Figure 1). The extra- cavity size was measured in the same perpendicular plane (Figure 1). For cavity distorting fibroids, we subjectively determined the plane of the endometrial- myometrial junction and measured the distance from this “imagined” ideal junction to the point of the fibroid furthest into the cavity in a perpendicular line (Figure 2). This was given a negative score, ensuring that an overlying endometrium was not included in the measurement. The size of extra- cavity component was also measured (Figure 2).

We calculated the protrusion ratio and expressed it as a percentage [A/(A+B) x 100] to determine the proportion of fibroid protrusion into the endometrial cavity. We calculated the mean diameter of each fibroid as the mean of diameter measurements taken in three orthogonal planes. We calculated fibroid surface area using the formula 4πr2, with r being radius of the fibroid and taken as a half of the mean of the diameter. We calculated the intra-cavitary surface area for submucosal fibroids as protrusion ratio x surface area. The age, parity and use of pre-operative gonadotrophin releasing-hormone analogue (GnRHa) was recorded for all patients. Endometrial cavity breaches at the time of surgery were identified from operation notes.

### Statistical Analysis

We examined demographic and ultrasonic variables for normality of distribution using the Kolmogorov – Smirnov test. Normally distributed variables were expressed as mean and standard deviation, whilst non - normally distributed variables were expressed as median and interquartile range. We performed univariate analysis using non - parametric statistics to compare medians and chi square test to compare proportions. We performed forward conditional logistic regression with breach of endometrial cavity as dependent variable and ultrasonic characteristics as dependent variables. Statistical significance was established at p<0.05 throughout all tests.

For internal validation, a 1000-fold bootstrapping was performed. We used SPSS ver. 24 and Stata ver. 15 for statistical analysis.

## Results

**Figure 3:**
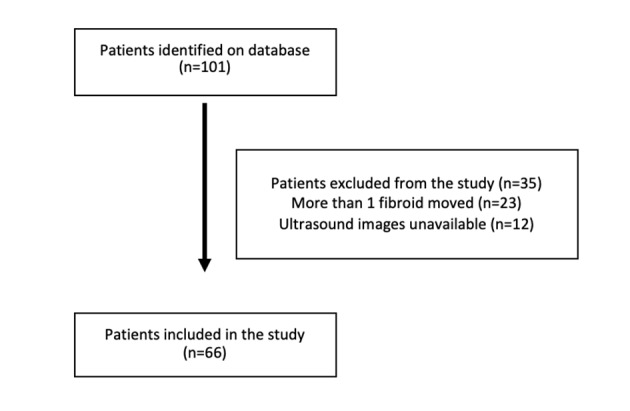
Flow chart of inclusion and exclusion of patients.

During the study period of December 2014 to October 2017, a total of 101 women underwent a laparoscopic myomectomy and were eligible to be included in the study. From these, 23 women were excluded as they had two or more fibroids removed and 12 women were excluded as it was not possible to obtain their pre-operative ultrasound images. Consequently, a final number of 66 women were included in the study (Figure 3). The main indication for myomectomy was heavy menstrual bleeding in 36/66 [54.5%; 95% CI 42.5 – 66.5], subfertility in 13/66 [20.0%; 95% CI 10.4 – 29.7], pressure symptoms in 10/66 [15.2%; 95% CI 6.5 – 23.9] and pelvic pain in 8/66 [12.1%; 95% CI 4.2 – 20.0].

Also, 40/66 (60.6%, 95% CI 48.8 – 72.4) women did not receive preoperative GnRHa. A cavity breach was sustained during 10/66 [15.1%, 95% CI 6.5 – 23.7] of the myomectomy procedures.

The ultrasound variables for the fibroids removed are shown in Table I. There was no significant difference between the median age of the women who had a cavity breach and those who did not [36 (IQR 32.75 – 40.25) vs 36 (IQR 32.0 – 39.75); p=0.572]. There was no significant difference in the proportion of women who sustained a cavity breach who were parous and those who were nulliparous [1/10 (10%, 95% CI 0.0 – 28.6) vs 15/56 (26.8%, 95% CI 15.2 – 38.4); p=0.239]. All breaches occurred in women who were not given preoperative GnRHa [10/40 (25.0%, 95% CI 11.6 – 38.4) vs. 0/26 (0.00%, 95% CI 0.00 – 0.00) p=0.005].

**Table I:** — Pre-operative ultrasound characteristics of all fibroids included in the study (n=66).

Ultrasonic variable	Median	IQR
Diameter (mm)	71.2	51.5 – 80.8
Minimum distance (mm)	7.5	-3.8 – 10.8
Surface area [SA] (mm^2^)	16375	8355 – 20869
Protrusion ratio (%)	0.00	0.00 – 5.275
Intracavity SA (mm^2^)	0.00	0.00 – 265.6
Extracavity size (mm)	70.2	51.4 – 79.0

Women who had a cavity breach had fibroids which were significantly smaller, closer to the cavity, with a greater intra-cavity surface area and smaller extra-cavity size than women who did not have a cavity breach (Table II). In a logistic regression model, protrusion ratio was selected as the best predictor of a breach (OR 1.22, 95% CI 1.10 – 1.37). The bootstrap adjusted OR was 1.22 (95% CI 1.03 – 1.46). The size of the fibroid, the intra-cavity surface area and extra-cavity size were not retained in the logistic regression model. The area under the curve for protrusion ratio to predict a breach was 0.91 (95% CI 0.79 – 1.0) with a cut off of 11% giving a sensitivity of 70% and specificity of 94.6%, positive likelihood ratio of 13.1 and negative likelihood ratio 0.31.

**Table II:** — Association between pre-operative ultrasound characteristics of fibroids and endometrial cavity breach at laparoscopic myomectomy.

Ultrasonic variable	Breach (n=10)	No breach (n=56)	p-value
Diameter (mm)	57.5 (38.0 – 71.65)	74.5 (58.00 – 83.2)	0.030
Minimum distance (mm)	-9.85 (-14.85 – -7.0)	8.6 (5.4 – 11.3)	0.001
Surface area [SA] (mm^2^)	10393 (4513 – 16225)	17671 (10699 – 22246)	0.020
Protrusion ratio (%)	17.1 (7.2 – 27.4)	0.00 (0.00 – 0.00)	0.001
Intracavity SA (mm^2^)	1463 (141 – 3304) 0.00	(0.00 – 0.00)	0.001
Extracavity size (mm)	54.0 (48.0 – 68.0)	72.8 (54.9 – 81.8)	0.001

## Discussion

Our study shows that a number of preoperative ultrasonic variables which appear to be predictive of endometrial cavity breach at the time of laparoscopic myomectomy. We found that protrusion ratio into endometrial cavity is the most effective variable with an optimal cut off of 11%. This information can be used to inform clinicians and to counsel patients. The rate of endometrial cavity breach in our study was 15.2% which is higher than the 6.1% reported by Dubuisson et al.(2000) and 3.4% by Dessolle et al.(2001). However, both these authors specifically excluded submucous fibroids from their studies. Given that we found that women with a significantly higher degree of fibroid protrusion are at higher risk of cavity breach, the rate difference between our study and others is likely due to differences in case selection.

The significance of cavity breach as an endpoint can be debated. To our knowledge there is no published data examining the relation between endometrial cavity breach after laparoscopic myomectomy and intrauterine adhesion formation. Some relevant information could be obtained from other surgical approaches to myomectomy.

Gupta et al. (2013) reported a 30% intrauterine adhesion formation in women who had a cavity breach at open myomectomy while in women having hysteroscopic myomectomy the rate of intrauterine adhesions is reportedly anywhere between 4.0% to 40% depending on technique and number of fibroids (Touboul et al., 2009; Yang et al., 2013; Mazzon et al., 2014). During hysteroscopic myomectomy opening the cavity happens as a matter of course and so it would appear reasonable to associate a cavity breach with increased risk of intrauterine adhesions. Based on this assumption it would be worth trying to identify patients at risk of a breach during laparoscopic myomectomy, and putting in place strategies to diminish that risk.

Ultrasonic assessment of women presenting with symptoms related to fibroids is routine and allows objective measurement of fibroid and uterine parameters (Stewart, 2015). Indeed, at the same time of avoiding a cavity breach at the time of laparoscopic myomectomy, it would possibly reduce the risk of intrauterine adhesion formation. Our study is the first to provide objective ultrasonic measurements to identify patients at increased risk of endometrial cavity breach at laparoscopic myomectomy. Surgeons operating on such patients should be more vigilant intra-operatively, adopting techniques to minimize the risk of a breach and, if such a breach occurs, the risk of intrauterine adhesions. Various approaches to such risk reduction have been proposed. Gupta et al. (2013) reported a reduced incidence of intrauterine adhesions with temporary placement of intrauterine Foley’s catheter filled with 30ml of normal saline in women who had endometrial cavity breach while Capmas et al. (2018) found a similar effect with the anti-adhesive gel Hyalobarrier ® (Gupta et al., 2013; Capmas et al., 2018). Mazzon et al. (2014) have suggested a technique for hysteroscopic fibroid resection focusing on minimisation of the use of electrocautery, lessons which are likely to apply to the laparoscopic approach. An alternative approach to transcervical resection of fibroids is presented by hysteroscopic morcellation. A recent systematic review concluded that this technique is equivalent to the more traditional technique in terms of risk of complications and post-operative adhesions (Vitale et al., 2017). Whether hysteroscopic morcellation can be routinely applied to myomas less than 50% intracavitary, i.e. type 2, remains to be explored. Such tumors may be better treated by ‘enucleation in toto’ or in two–steps as suggested by various authors (Pakrashi, 2014).

Our study is limited by its retrospective nature relying on stored images. Stored images may not have been acquired at the same point for each fibroid and thus lead to inaccurate assessment. The fact that the laparoscopic myomectomies were performed by four surgeons inevitably introduces variability. Finally, identification of a cavity breach is not standardized in our department, and thus, some breaches may have escaped unnoticed.

Despite its limitations, our study is the first to correlate pre-operative ultrasound findings with the risk of cavity breach, providing another dimension to pre-operative counselling in a relatively common procedure. Future directions of this work will be to define the risk of adhesion formation following laparoscopic myomectomy and examine whether cavity breach is indeed a risk factor for intrauterine adhesion formation.
